# Serum fibrinogen-to-albumin ratio predicts new-onset atrial fibrillation risk during hospitalization in patients with acute myocardial infarction after percutaneous coronary intervention: a retrospective study

**DOI:** 10.1186/s12872-023-03480-9

**Published:** 2023-09-01

**Authors:** Jiaqi Bao, Zhicheng Gao, Yilan Hu, Wenquan Liu, Lifang Ye, Lihong Wang

**Affiliations:** 1 The Second Clinical Medical College, Zhejiang Chinese Medical University, Hangzhou, 310053 People’s Republic of China; 2 Heart Center, Department of Cardiovascular Medicine, Zhejiang Provincial People’s Hospital (Affiliated People’s Hospital), Hangzhou Medical College, Hangzhou, Zhejiang, China; 3 Jinzhou Medical University, Jinzhou, Liaoning Province China

**Keywords:** Acute myocardial infarction, Atrial fibrillation, Serum fibrin-to-albumin ratio, Percutaneous coronary intervention, Inflammation, Coagulation, Fibrinolysis reaction

## Abstract

**Background:**

New-onset atrial fibrillation (NOAF) is a common adverse outcome of percutaneous coronary intervention (PCI) in patients with acute myocardial infarction (AMI) and is closely correlated with hospital stay and prognosis. In recent years, serum fibrinogen-to-albumin ratio (FAR), a novel biomarker for inflammation and thrombosis, has been used to predict the severity and prognosis of coronary artery disease. Our study aimed to investigate the relationship between FAR and NOAF during hospitalization after PCI in patients with AMI.

**Methods:**

We retrospectively analyzed the incidence of NOAF during hospitalization and follow-up in 670 patients with AMI after PCI. Data were collected on patient age, sex, body mass index, medical history, current medication, heart failure, laboratory tests, culprit blood vessels, echocardiographic characteristics, and AMI type. The enrolled patients were divided into NOAF and non-NOAF groups. The baseline characteristics of patients in the two groups were compared, and the predictive correlation between FAR and NOAF was evaluated using logistic regression analysis and the receiver operating characteristic curve.

**Results:**

Fifty-three (7.9%) patients developed NOAF during hospitalization. The occurrence of NOAF was found to be independently associated with higher FAR besides older age, higher neutrophil count, greater left atrial size, worse Killip class upon admission, lower body mass index, lower platelet count, lower left ventricle ejection fraction, and target left circumflex artery disease. FAR exhibited a better predictive value for the occurrence of NOAF during hospitalization (area under the curve, 0.732; 95% confidence interval, 0.659–0.808).

**Conclusions:**

FAR is a robust tool for predicting NOAF risk during hospitalization in patients with AMI after PCI and has a better predictive value than serum fibrin and serum albumin levels alone.

**Supplementary Information:**

The online version contains supplementary material available at 10.1186/s12872-023-03480-9.

## Background

New-onset atrial fibrillation (NOAF) is a common complication of acute myocardial infarction (AMI), with an incidence rate of 6–21% [1] that is related to the prolongation of hospital stay, and an increase in the incidence and mortality of adverse cardio-cerebrovascular disease [2, 3]. Although it is not yet clear whether NOAF is a causative factor or a concomitant manifestation of poor prognosis, it is a risk factor for a deterioration of prognosis during hospital stay [4]. Therefore, early identification of patients with AMI having a higher risk of developing NOAF is crucial for the effective management of these cases. The specific mechanism of NOAF in patients with AMI after percutaneous coronary intervention (PCI) is complex and unclear, and is influenced by various risk factors, including advanced age, female sex, and heart failure. However, it is mainly associated with coronary artery blood flow disorders, embolic effects, and inflammatory reactions [5, 6]. At present, reliable and easy-to-assess clinical predictors for the early identification of NOAF are lacking. Therefore, a better understanding of the risk factors for the occurrence and development of NOAF after PCI will benefit patients with AMI by making preventive measures possible.

Fibrinogen (FIB) is a soluble glycoprotein synthesized mainly in the liver that participates in platelet aggregation and activation, smooth muscle cell proliferation, and the expression of cell adhesion molecules and pro-inflammatory factors [7, 8]. It plays a central role in coagulation cascades, inflammation, and the process of coronary atherosclerosis [9, 10]. As an acute reactant, the FIB concentration increases in the inflammatory state. Many observational studies have found that plasma FIB levels are independently associated with all-cause mortality and long-term prognosis in patients with coronary artery disease (CAD) after PCI [11, 12]; however, there are no reports to the short-term effects of FIB levels.

Albumin (ALB) is the most abundant protein in human extracellular fluid. It not only has anti-inflammatory and antioxidant effects but can also inhibit platelet aggregation and activation to affect plasma viscosity [13]. Low ALB levels are associated with adverse cardiac events in patients with CAD after PCI [14].

FIB and ALB levels are negatively correlated with each other and are important mediators of changes in blood flow and inflammatory. Low ALB levels act as a compensatory response by stimulating the synthesis of lipoproteins and procoagulative factors (such as factor V and VIII and FIB), resulting in hyperlipidemia and a hypercoagulable state, ultimately promoting atherosclerotic plaque formation and thrombosis [15]. The serum fibrinogen-to-albumin ratio (FAR), combines the above mentioned two indices, is independently related to the severity and long-term prognosis of CAD, and can be used by clinicians to improve the risk stratification of patients with AMI post-PCI [16]. This study speculated that FAR could supplement the disease dimensions missing from the GRACE score that are related to the outcomes of patients with AMI, such as inflammation, blood flow status, and CAD. More importantly, FAR is a novel inflammatory marker similar to the C-reactive protein-to-albumin ratio (CAR). We have extensively studied the predictive value of CAR in cardiovascular related patients. Whether in patients with coronavirus disease 2019 [17] or those with heart failure who received implantable cardiac defibrillator treatment and had a reduced ejection fraction [18], high CAR levels are known to increase in-hospital mortality and the long-term risk of death.

We speculate that combining FIB and ALB into a single index (FAR) may be more sensitive and specific than using FIB or ALB alone to predict the risk of NOAF. The purpose of our study was to evaluate whether FAR could be used as an early circulatory biomarker for predicting the risk of NOAF and for the risk stratification of patients with AMI.

## Methods

### Study design and patient population

This single-center, retrospective, observational cohort study was conducted between January 2020 and December 2022 at the Zhejiang Provincial People’s Hospital (Hang Zhou, China). We collected data from 723 patients with AMI with no history of atrial fibrillation (AF) who underwent their first PCI during hospitalization. Our diagnostic standards for AMI were in accordance the European Society of Cardiology/American College of Cardiology criteria [19], including ST-segment elevation myocardial infarction (STEMI) and non-STEMI that was defined as AF during hospitalization after PCI. During hospitalization, AF episodes were monitored and recorded by a 12-lead electrocardiogram (ECG) or a Holter monitoring device (duration ≥ 30 s).

**Fig. 1:**
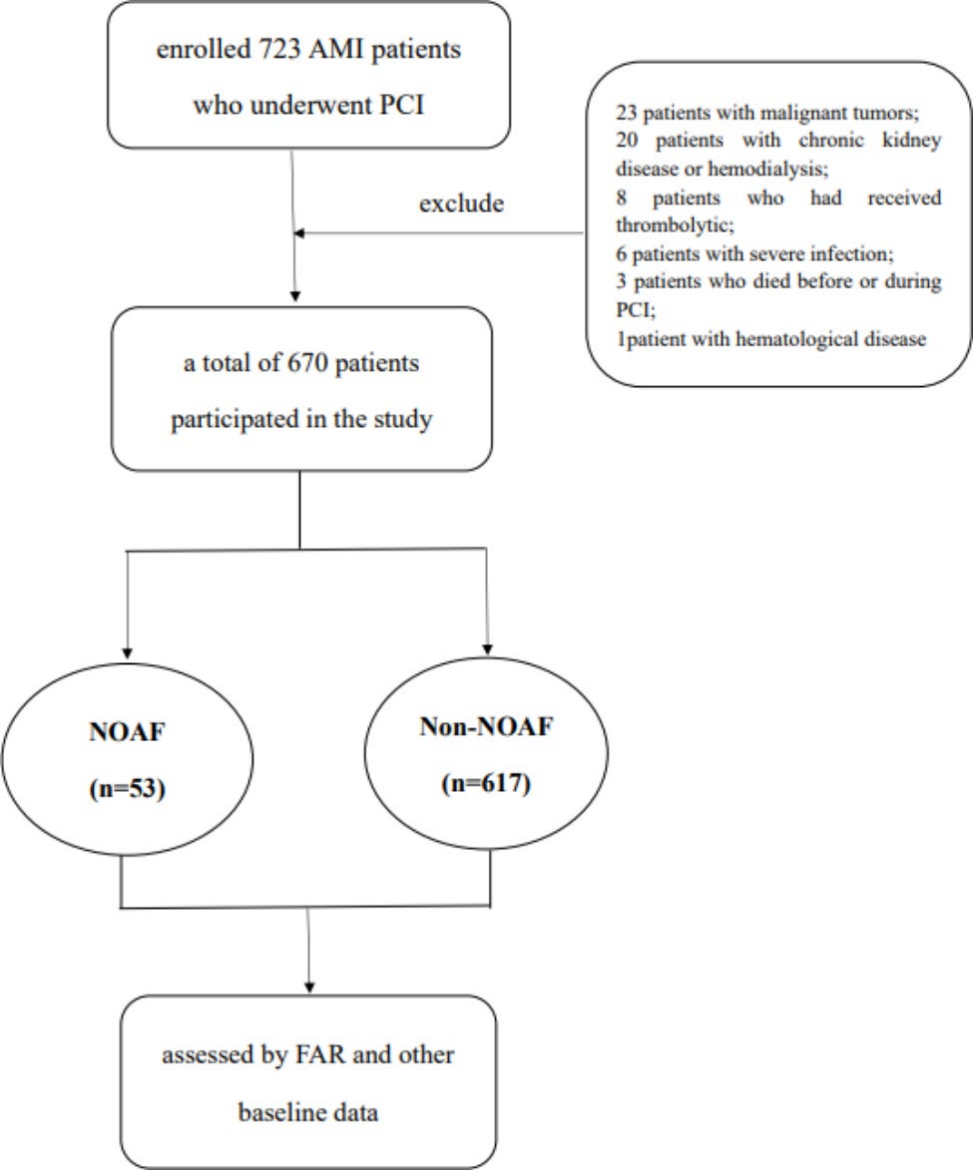
Flow chart of the population. AMI, acute myocardial infarction;PCI, percutaneous coronary intervention; NOAF, New-onset Atrial Fibrillation; FAR, Serum Fibrin to Serum Albumin ratio

We excluded patients who [1] had pre-existing AF or AF presented at admission; [2] had undergone thrombolytic therapy or emergent coronary artery bypass grafting (CABG) surgery; [3] had severe cardiac valve disease or congenital heart disease; [4] had chronic kidney disease or nephrotic syndrome, severe liver dysfunction, or cirrhosis; [5] had a chronic consumptive disease such as a malignant tumor, tuberculosis, hyperthyroidism, severe infection, hematological disease, etc.; [6] died before or during PCI; and [7] had a history of surgery up to 2 weeks before the current hospitalization. We recruited 723 patients with AMI who underwent PCI. We excluded 23 patients with malignant tumors, 20 with chronic kidney disease or hemodialysis, 8 who had received thrombolytic therapy, 6 with severe infection, 3 who died before or during PCI, and 1 with hematological disease. Ultimately, 670 patients were included in the study (Fig. 1). This study was reviewed and approved by the Human Ethics Committee of Zhejiang Provincial People’s Hospital (Hangzhou, China; Approval Number: QT2023257). The requirement of obtaining informed consent from patients was waived by the hospital review board due to the retrospective nature of our study. At the same time, patient data were anonymized or maintained with confidentiality.

### Data collection

We obtained the following data from the medical records of patients in our hospital database: baseline patient characteristics (age, sex, height, weight, body mass index [BMI]), vital signs on admission (heart rate, systolic and diastolic blood pressure), presence of complications (hypertension, diabetes, stroke, coronary artery disease), current smoking and drinking status, current drug use, laboratory test results before heparin administration or the commencement of the reperfusion process (blood cell counts for white blood cells [WBCs], lymphocytes, monocytes, neutrophils, erythrocytes, and platelets; serum levels of high-sensitivity C-reactive protein, FIB, ALB, total cholesterol [TC], triglycerides [TG], high-density lipoprotein cholesterol [HDL-C], low-density lipoprotein cholesterol [LDL-C], lipoprotein (a), uric acid, creatinine [Scr], and B-type natriuretic peptide [BNP]; estimated glomerular filtration rate [eGFR]; and cardiac troponin I levels), echocardiography results (left atrial diameter [LA] and left ventricular ejection fraction [LVEF]) type of AMI, type of coronary artery stenosis, and Killip class. The normal serum FIB range was 2.00–4.00 g/L, whereas the normal serum ALB range was 4.00–5.50 g/dL. FAR was calculated by dividing the serum FIB (g/L) value by the serum ALB (g/dL) value. All patients underwent coronary angiography via the femoral or radial route. Coronary artery stenosis is defined as stenosis ≥ 50% in any coronary artery (including the left main artery [LM], left anterior descending artery [LAD], left circumflex artery [LCX], and right coronary artery [RCA] observed by coronary angiography. Three-vessel CAD was defined as coronary angiography confirming that all three main coronary arteries (LAD, LCX, RCA) have stenosis ≥ 50%. During the physical examination, the patients were divided into four classes according to the Killip classification: class I, no signs of heart failure; class II, left heart failure with lung rales occupying < 50% of the lung field; class III, acute pulmonary edema; and class IV, cardiogenic shock with varying degrees of hemodynamic changes. Transthoracic echocardiography was performed by an experienced ultrasound physician within 24 h of patient admission. Anticoagulant and antiplatelet medications were administered to patients in accordance with current AMI guidelines. Patients routinely received antiplatelet agents including 300 mg of aspirin and a purinergic receptor P2Y, G-protein coupled, 12 protein (P2Y12) inhibitor (clopidogrel 300-600 mg, ticagrelor 180 mg, or prasugrel 60 mg) before the procedure, followed by daily aspirin (100 mg) and P2Y12 inhibitors (clopidogrel 75 mg once, ticagrelor 90 mg twice, or prasugrel 10 mg once daily).

### Follow-up

Our AF diagnosis was consistent with the consensus guidelines [20]. Telemetric ECG (standard 12-lead) was performed once per day until discharge to monitor all patients while they remained hospitalized in the cardiac care unit following PCI. All patients also completed a 24-h Holter monitoring after surgery. The follow-up endpoint was patient discharge. The electronic monitoring data of all participants were reviewed to determine the occurrence of NOAF. NOAF was defined as the detection of AF lasting ≥ 30 s during post-PCI hospitalization.

### Statistical methods

Statistical analyses were performed using IBM SPSS Statistics software (version 26.0; SPSS Inc. Corp., Armonk, NY). Normally distributed continuous variables are presented as means and standard deviations, whereas non-normally distributed data are presented as medians with interquartile ranges. The Kolmogorov–Smirnov test was used to analyze normally distributed data. The Mann–Whitney *U*-test was used to analyze non-normally distributed data, while Student’s *t*-test was used to analyze normally distributed data. Categorical data are described as frequencies (percentages) and were analyzed using Pearson’s chi-square test. Univariate and multivariate logistic regression models were used to determine the relationship between the variables and NOAF. We conducted a multicollinearity test on potential risk factors determined by the univariate analysis, selected variables with a variance expansion factor (VIF) < 3, and incorporated more meaningful variables into the multivariate analysis according to experience to determine independent risk factors related to NOAF after PCI. To determine the predictive performance of FAR for NOAF after PCI, we used receiver operating characteristic (ROC) curves. We further evaluated the predictive performance of FAR through a subgroup analysis of AMI. In addition, we divided FAR into quartiles for the Mantel–Haenszel chi-squared test to evaluate whether there was a linear relationship with the incidence rate of NOAF. Pearson’s correlation analysis was used to investigate the correlations between two variables. For logistic regression analysis, the median quartile was used as a quasi-continuous variable to calculate the P value of the trend. Statistical significance was defined as a two-sided P-value < 0.05.

## Results

### Baseline patient characteristics

Table 1 summarizes the baseline characteristics of this patient cohort. A total of 670 patients with AMI were included in this study, including 324 (48.4%) STEMI and 346 (51.6%) non-STEMI patients. The average age was 61 years, and 563 (84%) were men. Fifty-three (7.9%) patients developed NOAF during hospitalization. There was no significant difference between the two groups in terms of sex distribution, height, heart rate, systolic blood pressure, current smoking or drinking status, medications, history of hypertension, history of coronary artery disease, blood counts of lymphocytes, monocytes, serum levels of high-density lipoprotein cholesterol, lipoprotein (a), uric acid, or the type of AMI. Compared with the patients in the non-NOAF group, those in the NOAF group were older and more likely to have a higher FAR; blood cell counts for WBCs and neutrophils; serum levels of high-sensitivity C-reactive protein, FIB, Scr and BNP; cardiac troponin I; LA; and Killip class, as well as a history of diabetes or stroke (p<0.05). Patients in the NOAF group had a lower body weight, BMI, diastolic blood pressure, blood cell counts for erythrocytes, platelets, serum levels for ALB, total cholesterol, triglycerides, low-density lipoprotein cholesterol, and eGFR and LVEF values (p<0.05). As for the angiography results, patients in the NOAF group were more likely to have three-vessel disease, and target lesions in RCA and LCX (p<0.05).

**Table 1:** Baseline characteristics of study population based on the absence or presence of NOAF

Variables			NOAF n = 53		Non-NOAF n = 617		P-value
Demographic characteristics							
Male, n(%)			44(83%)		519(84.1%)		0.834
Age,(y)			70.6 ± 12.82		60.54 ± 13.17		<0.001
Height(cm)			166.49 ± 7.13		167.78 ± 6.9		0.190
Weight(kg)			64.31 ± 9.32		70.49 ± 11.34		<0.001
BMI(kg/m2)			23.14 ± 2.55		24.96 ± 3.20		<0.001
Heart rate at admission(bpm)			80(70.5–95)		78(70–89)		0.155
SBP(mmHg)			131.37 ± 28.97		138.47 ± 26.07		0.059
DBP(mmHg)			76.19 ± 15.56		83.15 ± 17.17		0.004
Current smoker, n(%)			21(39.6%)		299(48.5%)		0.217
Current drinker, n(%)			16(30.2%)		189(30.6%)		0.946
**Medications**							
ACEI/ARB, n(%)			12(22.6%)		136(22%)		0.920
Beta-blocker, n(%)			5(9.4%)		48(7.8%)		0.669
Calcium-blocker, n(%)			24(45.3%)		210(34%)		0.100
Statin, n(%)			5(9.4%)		60(9.7%)		0.945
Aspirin, n(%)			6(11.3%)		63(10.2%)		0.802
Clopidogrel, n(%)			4(7.5%)		37(6%)		0.652
**Comorbidities**							
Hypertension, n(%)			37(69.8%)		354(57.5%)		0.080
Diabetes, n(%)			19(35.8%)		137(22.2%)		0.024
History of stroke, n(%)			7(13.2%)		36(5.8%)		0.036
History of coronary heart disease, n(%)			4(7.5%)		37(6%)		0.946
**Laboratory tests**							
WBC (10^9^/L)			9.83(8.39–12.90)		8.67(6.72–11.16)		0.001
Lym (10^9^/L)			1.40(1.03–2.07)		1.70(1.24–2.28)		0.061
Mon (10^9^/L)			0.50(0.40–0.69)		0.45(0.33–0.60)		0.166
Neu (10^9^/L)			7.6(5.50–10.50)		5.85(4.28–8.40)		<0.001
RBC (10^9^/L)			4.56(4.02–4.75)		4.64(4.25–5.02)		0.042
Plt (10^9^/L)			181(139.50–210)		207(175–248)		<0.001
Hs-CRP(mg/L)			7.70(2.00-56.55)		2.80(1.30–7.25)		<0.001
Serum fibrinogen(g/L)			4.27(3.025–5.685)		3.010(2.530–3.750)		<0.001
Serum albumin(g/dL)			3.47(3.28–3.71)		3.74(3.55–3.95)		<0.001
FAR(10^− 3^)			1.226(0.826–1.558) 0.796(0.659–1.005)				<0.001
TC (mmol/L)			4.30(3.57–5.11)		4.58(3.96–5.36)		0.041
TG (mmol/L)			1.06(0.77–1.61)		1.49(1.08–2.15)		<0.001
HDL-C (mmol/L)			0.95(0.80–1.07)		0.93(0.81–1.07)		0.797
LDL-C (mmol/L)			2.64(2-3.27)		2.86(2.27–3.47)		0.040
Lipoprotein(a) (mg/L)			175(108-377.5)		180.5(91.25–330)		0.716
Uric-acid (umol/L)			362(316.50–460)		358(288.5–434)		0.163
Scr (umol/L)			93.7(81.25–107)		80.8(71.8-92.25)		<0.001
eGFR (mL/min*1.73m^2^)			72.75 ± 27.26		89.84 ± 22.19		<0.001
BNP (pg/mL)			400.2(155.8-1075.05)119.10(46.70-266.60)				<0.001
cTnI (ug/L)			4.35(0.48–16.21)		0.99(0.16–5.82)		0.004
**Echocardiography results**							
LA (mm)			40.68 ± 4.51		37.19 ± 4.54		<0.001
LVEF (%)			50(41.5–58)		60(53–65)		<0.001
**Type of AMI, n (%)**							
STEMI			32(60.4%)		292(47.3%)		0.068
NSTEMI			21(39.6%)		325(52.7%)		0.068
**Coronary artery stenosis>50%, n (%)**							
LM			1(1.9%)		44(7.1%)		0.144
LAD			46(86.8%)		548(88.8%)		0.656
LCX			45(84.9%)		379(61.4%)		0.001
RCA three-vessel disease			44(83%) 35(66.0%)		422(68.4%) 279(45.2%)		0.027 0.004
**Killip class, n (%)**							
I			16(30.8%)		481(78%)		<0.001
II			23(44.2%)		87(14.1%)		<0.001
III			8(15.1%)		21(3.4%)		<0.001
IV			8(15.1%)		28(4.5%)		0.001

**Abbreviations**: NOAF, New-onset Atrial Fibrillation; BMI, Body mass index; SBP, Systolic blood pressure; DBP, Diastolic blood pressure; ACEI, Angiotensin converting enzymeinhibitors; ARB, Angiotensin receptor blocker; WBC, White blood cell count; Lym, Lymphocyte count; Mon, Monocyte count; Neu, Neutrophil count; RBC, Red blood cell count; Plt, platelet count; Hs-CRP, High-sensitivity C-reactive protein, FAR, Fibrin/Albumin ratio; TC, Total cholesterol; TG, triglyceride; HDL-C, High-density lipoprotein cholesterol; LDL-C, Low density lipoprotein cholesterol; Scr, Serum creatinine; eGFR, Estimated glomerular filtration rate; BNP, B-type natriuretic peptide; cTnI, cardiac troponin I;LA, Left atrium; LVEF, left ventricular ejection fraction; AMI, Acute myocardial infarction; STEMI, ST segment elevation myocardial infarction; NSTEMI, Non ST segment elevation myocardial infarction; LM, Left main coronary artery; LAD, Left anterior descending branch; LCX, Left circumflex branch; RCA, Right coronary artery

### Clinical predictors of incident NOAF

We included numerous variables from both groups of patients in the univariate regression analysis, and the results showed that the following variables were significantly associated with the occurrence of NOAF (Table 2): patient age (odds ratio [OR], 1.061; 95% confidence interval [CI], 1.037–1.085, P < 0.001), FAR (OR, 4.299; 95% CI, 2.537–7.286, P < 0.001), and WBC count (OR, 1.119; 95% CI, 1.043–1.201, P = 0.002). Prior to conducting a multivariate analysis, multicollinearity analysis showed that there was multicollinearity between patient body weight and BMI, WBC count and Neu, FAR and FIB or ALB, Scr and eGFR, three-vessel disease and target lesions in the RCA or LCX, and the four classes of Killip with a variance inflation factor (VIF) > 3. Consequently, we selected BMI, Neu, FAR, eGFR, target lesions in the RCA, target lesions in the LCX, Killip class > 2, and other variables in the multivariate analysis (Table 2). These variables had a VIF < 2. After adjusting for confounding factors, we found that high FAR is an independent predictor of NOAF occurrence in patients with AMI after PCI (OR, 3.377; 95% CI, 1.562–7.298, P = 0.002). In addition, based on the median time of onset of NOAF (2 days), we divided patients with NOAF into two subgroups: those with an onset within 2 days and those with an onset later than 2 days. This analysis showed no significant difference in FAR between the two subgroups. We speculate that, based on its magnitude, FAR can predict the onset of NOAF but not the timing of onset. Additionally, our multivariate logistic regression analysis showed other significant predictors, including patient age (OR, 1.045; 95% CI, 1.007–1.084, P = 0.019), BMI (OR, 0.843; 95% CI, 0.733–0.969, P = 0.016), Neu (OR, 1.150; 95% CI, 1.033–1.280, P = 0.011), platelet count (Plt) (OR, 0.991; 95% CI, 0.985–0.997, P = 0.003), LA (OR, 1.104; 95% CI, 1.023–1.191, P = 0.011), LVEF (OR, 0.964; 95% CI, 0.931–0.998, P = 0.041), LCX (OR, 2.984; 95% CI, 1.155–7.711, P = 0.024), and Killip class>2 (OR, 3.073; 95% CI, 1.310–7.208, P = 0.010).

**Table 2:** Predictors of NOAF variable univariate analysis multivariate analysis

Variables			OR			95%CI				P
Univariate analysis										
Age,(y)			1.061			1.037–1.085				<0.001
Weight(kg)			0.948			0.923–0.975				<0.001
BMI(kg/m^2^)			0.816			0.738–0.902				<0.001
DBP(mmHg)			0.974			0.957–0.992				0.005
Diabetes, n(%) History of stroke, n(%)			1.958 2.456			1.082–3.541 1.036–5.824				0.026 0.041
WBC (10^9^/L)			1.119			1.043–1.201				0.002
Neu (10^9^/L)			1.131			1.053–1.215				0.001
RBC (10^9^/L)			0.562			0.358–0.884				0.013
Plt (10^9^/L)			0.991			0.986–0.996				0.001
Hs-CRP(mg/L)			1.013			1.008–1.019				<0.001
Serum fibrinogen(g/L)			1.657			1.377–1.994				<0.001
Serum albumin(g/dL)			0.132			0.060–0.294				<0.001
FAR(10^− 3^)			4.299			2.537–7.286				<0.001
TC (mmol/L)			0.799			0.616–1.036				0.091
TG (mmol/L)			0.719			0.518–0.999				0.049
LDL-C (mmol/L)			0.709			0.508–0.991				0.044
Scr (umol/L)			1.016			1.009–1.024				<0.001
eGFR (mL/min*1.73m^2^)			0.970			0.958–0.982				<0.001
BNP (pg/mL)			1.001			1.001–1.001				<0.001
cTnI (ug/L)			1.005			0.998–1.012				0.133
LA (mm)			1.155			1.089–1.226				<0.001
LVEF (%)			0.936			0.913–0.960				<0.001
LCX (%)			3.532			1.637–7.624				0.001
RCA (%) three-vessel disease (%)			2.259 2.356			1.081–4.720 1.306–4.250				0.030 0.004
Killip I (%)			0.126			0.068–0.233				<0.001
Killip II (%)			4.832			2.672–8.737				<0.001
Killip III (%)			5.046			2.116–12.029				<0.001
Killip IV (%)			3.740			1.611–8.681				0.002
**Multivariate analysis**										
Age,(y) BMI(kg/m2) Neu (10^9^/L)			1.045 0.843 1.150			1.007–1.084 0.733–0.969 1.033–1.280				0.019 0.016 0.011
Plt (10^9^/L)			0.991			0.985–0.997				0.003
FAR(10^− 3^) LA (mm) LVEF (%)			3.377 1.104 0.964			1.562–7.298 1.023–1.191 0.931–0.998				0.002 0.011 0.041
LCX (%)			2.984			1.155–7.711				0.024
Killip>II (%)			3.073			1.310–7.208				0.010

NOAF, New-onset Atrial Fibrillation; BMI, Body mass index; DBP, Diastolic blood pressure; WBC, White blood cell count; Neu, Neutrophil count; RBC, Red blood cell count; Plt, platelet count; Hs-CRP, High-sensitivity C-reactive protein, FAR, Fibrin/Albumin ratio; TC, Total cholesterol; TG, triglyceride; LDL-C, Low density lipoprotein cholesterol; Scr, Serum creatinine; eGFR, Estimated glomerular filtration rate; BNP, B-type natriuretic peptide; cTnI, cardiac troponin I;LA, Left atrium; LVEF, left ventricular ejection fraction; LCX, Left circumflex branch; RCA, Right coronary artery

### FAR: a good predictor of NOAF after PCI in patients with AMI

**Fig. 2:**
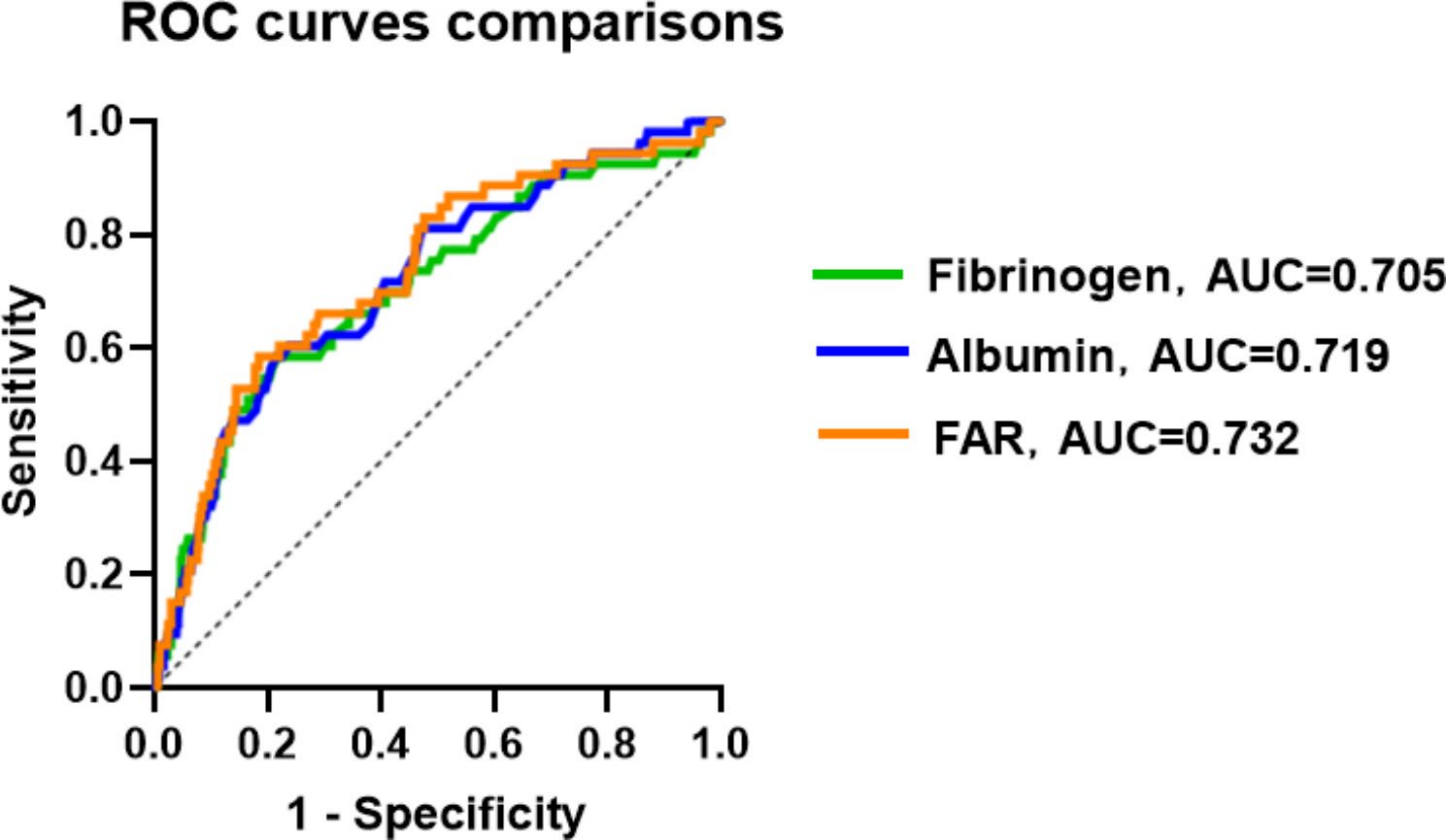
Receiver operating characteristic curves of Serum fibrinogen-to-albumin ratio (FAR), fibrinogen, albumin

**Fig. 3:**
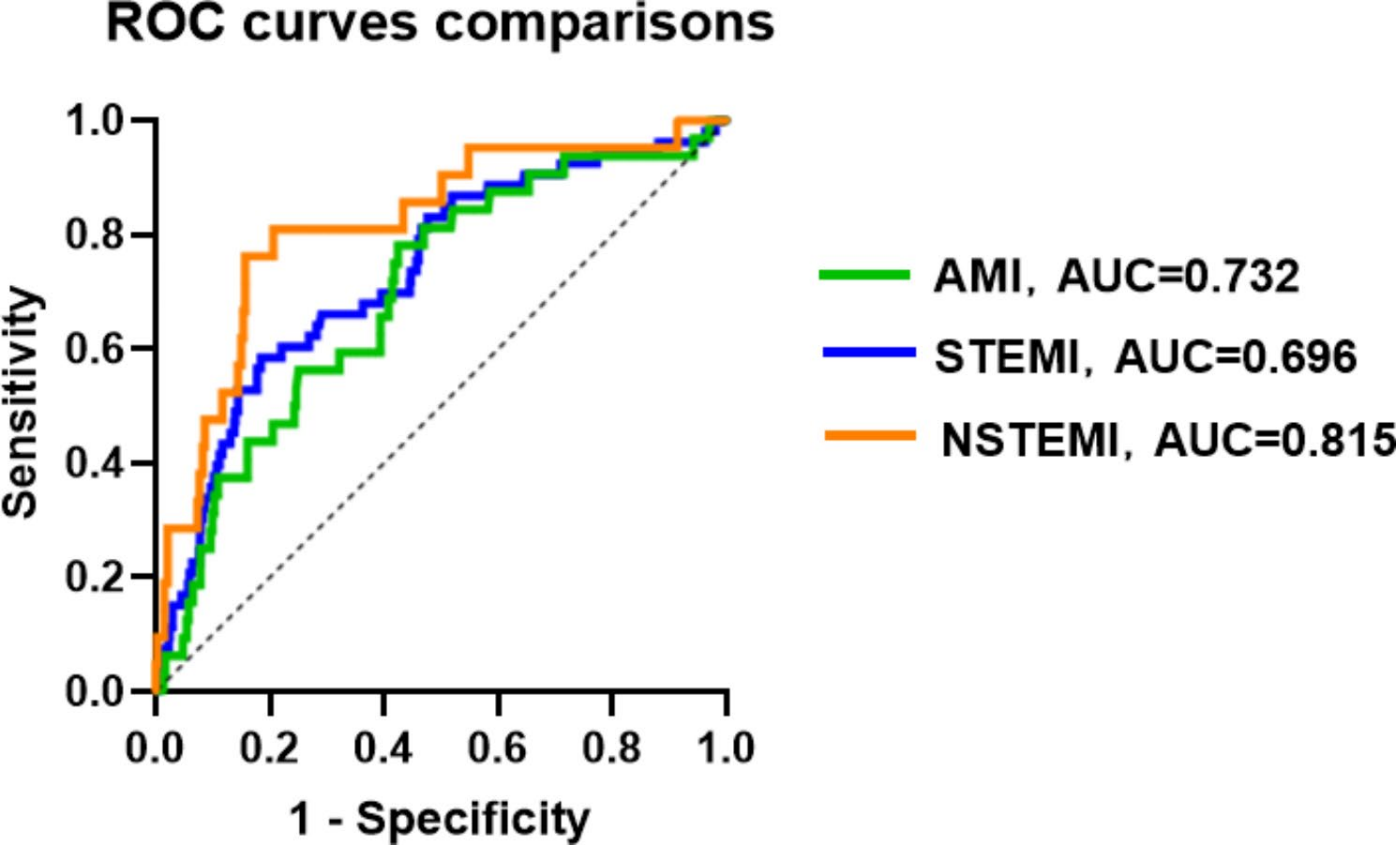
Receiver operating characteristic curves of each subgroup of acute myocardial infarction (AMI). STEMI, ST segment elevation myocardial infarction; NSTEMI, Non-ST segment elevation myocardial infarction

The ROC of FAR (as shown in Fig. 2) can accurately predict the incidence rate of NOAF during hospitalization in patients with AMI post-PCI (area under the curve [AUC], 0.732; 95% CI, 1.562–7.298, P<0.001). A FAR value>1.142 distinguished patients at risk for NOAF with 58.5% sensitivity and 81.5% specificity (maximum Youden index J, 0.400). The predictive role of FAR was significantly superior to that of FIB (AUC, 0.705; 95% CI, 0.626–0.783) or ALB (AUC, 0.719; 95% CI, 0.646–0.792), as shown in Fig. 2. At the same time, using the classification of AMI, we analyzed the ROCs of the two subgroups (as shown in the Fig. 3), and FAR exhibited a strong predictive ability in the NSTEMI subgroups (AUC, 0.815; 95% CI, 0.716–0.914). An FAR>0.815 distinguished patients at risk of NOAF with 76.2% sensitivity and 84.3% specificity. However, FAR showed only an average predictive ability in the STEMI subgroup (AUC, 0.696; 95% CI, 0.620–0.791). In addition, we conducted an ROC analysis on patient age, BMI, Neu, Plt, LA and LVEF that represented other continuous independent predictors. However, their predictive powers were poorer than that of FAR, with AUC values of 0.719 (95% CI, 0.646–0.792) for age, 0.659 (95% CI, 0.590–0.727) for BMI, 0.653 (95% CI, 0.581–0.725) for Neu, 0.656 (95% CI, 0.579–0.733) for Plt, 0.710 (95% CI, 0.640–0.799) for LA, and 0.729 (95% CI, 0.660–0.798) for LVEF (Table 3).

**Table 3:** Independent continuous predictors: AUC, CI, cutoff value, relative sensitivity, and specificity

Predictors	AUC	95%CI	Cutoff value		Sensitivity, %		Specificity, %	
FAR								
AMI	0.732	0.659–0.808	1.142		58.5%		81.5%	
STEMI	0.696	0.602–0.791	0.816		78.1%		57.5%	
NSTEMI	0.815	0.716–0.914	1.219		76.2%		84.3%	
Fib	0.705	0.626–0.783	3.885		58.5%		78.3%	
Alb	0.719	0.646–0.792	3.515		58.5%		78.4%	
Age	0.719	0.646–0.792	70.5		60.4%		78.1%	
BMI Neu Plt LA LVEF	0.659 0.653 0.656 0.710 0.729	0.590–0.727 0.581–0.725 0.579–0.733 0.640–0.799 0.660–0.798	25.74 6.715 212.5 36.5 54.4		62.2% 67.9% 79.2% 84.9% 69.8%		73.5% 61.4% 46.4% 46.8% 72.1%	

**Abbreviations**: AUC, area under the curve; CI, confidence interval; FAR, Fibrin/Albumin ratio; AMI, Acute myocardial infarction; STEMI, ST segment elevation myocardial infarction; NSTEMI, Non-ST segment elevation myocardial infarction; Fib, Serum fibrinogen; Alb, Serum albumin; BMI, Body mass index; Neu, Neutrophil count; Plt, platelet count; LA, Left atrium; LVEF, left ventricular ejection fraction

We then divided FAR into quartiles to evaluate whether there was a linear relationship between FAR and NOAF during hospitalization in patients with AMI post-PCI. Patients with FAR values ≥ 1.07 had an incidence rate of 18.56%, compared to 2.40% for those with FAR values < 0.67, 3.57% for those with FAR values ranging between 0.67 and 0.82, and 7.14% for those with FAR values ranging between 0.82 and 1.07 (P < 0.001 for trend). These findings were in agreement with our hypothesis that the magnitude of -FAR was associated with an increased NOAF risk (Supplementary material).

## Discussion

In the present study, we evaluated independent risk factors for NOAF during hospitalization in patients with AMI post-PCI. These included older age, higher neutrophil count, FAR, LAD, worse Killip class, lower BMI, platelet count, LVEF, and concomitant LCX disease. AMI type was not found to be a risk factor. More importantly, our findings revealed that higher serum FAR values have a better predictive power for NOAF after PCI in terms of sensitivity (58.5%) and specificity (81.5%) with a C-statistic of 0.732 (Table 3), even after adjustment, especially in NSTEMI patients (AUC = 0.815; 95% CI, 0.716–0.914, sensitivity, 76.2%; specificity, 84.3%).

NOAF is a common outcome in patients with AMI after PCI. In our present study, we found that 7.9% of patients experienced AF during hospitalization, and this is consistent with previous studies [21].

Previous studies have shown that NOAF after revascularization in patients with AMI is related to longer hospital stays, higher mortality rates, and poorer prognoses (including ischemic stroke, reinfarction, and heart failure) [22, 23]. In the cohort of this study, patients in the NOAF group had a longer hospital stay, with a median hospital stay of 10 days, whereas those in the non-NOAF group had a median hospital stay of 6 days that was statistically significant. Adverse outcomes in patients with AMI are related to the adverse hemodynamic effects caused by NOAF. The loss of effective atrial contraction and atrioventricular synchronization, acceleration of the ventricular rate, valve regurgitation, and irregularity of the beat-to-beat interval, all lead to a reduction in cardiac output [24]. Therefore, early identification of patients at high risk of NOAF after PCI is clinically valuable for ensuring preventive treatment and care during hospitalization.

The etiology of NOAF is multifactorial and complex, including coronary artery blood flow disorders, embolic effects, atrial ischemia or infarction, local and systemic inflammation, and hormone activation [6, 25, 26]. These events then serve as triggering factors or participants in the process of arrhythmia onset, leading to structural and electrical remodeling of the atrium and contributing to NOAF. The occurrence of AF further stimulates these pathophysiological pathways through a positive feedback loop, commonly known as “AF producing AF” [27]. Excessive atrial traction caused by acute heart failure after myocardial infarction may increase atrial excitability and the lengths of conduction pathways [28]. Infarction-related pericarditis has been described as a direct cause of AF [29].

Advanced age is an independent risk factor for AF [30]. Numerous studies have reported that older individuals are more likely to develop NOAF after AMI [3, 31, 32]. Our current data also demonstrate that advanced age is an independent risk factor for NOAF after PCI, demonstrating good predictive value.

A meta-analysis showed that obesity increases the incidence rate of AF in the general population, and the risk increases with an increase in BMI [33]. However, some researchers have proposed the “obesity paradox” in the CAD patient population, where overweight or obese patients have a lower risk of hospital complications and 1-year mortality than normal and lean patients [34], as well as a lower risk of adverse heart events and stroke [35]. Our present data also agree with the “obesity paradox” of patients with CAD and show that patients with a lower BMI are more likely to develop AF. Adiponectin produced in the adipose tissue is protective for atherosclerosis, and this may improve myocardial ischemia status and reduce AF risk in patients with AMI [36]. Adipose tissue can also produce soluble tumor necrosis factor receptors to neutralize the harmful effects of tumor necrosis factor-α expression on the myocardium [37].

Hyperlipidemia is a recognized risk factor for CAD, and lipid-lowering therapy is the cornerstone of treatment for patients with CAD [38, 39]. However, paradoxically, our study found a negative correlation of TC, LDL-C, and TG with NOAF that has also been reported in previous studies [40, 41]. Although the exact mechanism of this contradictory association is still unclear, cholesterol consumption increases the intracellular Ca^2+^concentration and triggers signal cascade reactions, ultimately leading to myofibrillar destruction and cardiomyocyte contraction disorders [42]. This may partly explain the higher incidence of NOAF in patients with AMI and lower lipid levels.

Killip grading is a simple clinical tool used to assess the severity of heart failure in patients with AMI. A meta-analysis indicated that a higher Killip class after AMI has a strong predictive value for NOAF [21]. In our study, we found that patients in the NOAF group had a higher Killip class, larger left atrium, and lower ejection fraction. A low ejection fraction is also associated with contrast agent nephropathy after PCI [43]. An increase in the left atrial diameter is a sign of left atrial myocardial dilation and remodeling and is the basis for triggering and maintaining AF [44]. The acute increase in atrial filling pressure and left ventricular dysfunction are related to the occurrence of NOAF [45]. Similarly, animal studies have shown that elevated atrial pressure leads to a significant increase in the susceptibility to AF [46].

In most patients, the LCX is the smallest epicardial vessel that supplies the ventricular myocardium. When LCX lesions occur, their appearance on the electrocardiogram is usually not easily visible i.e., there is no ST-segment elevation [47]. Therefore, patients with acute coronary syndrome caused by LCX coronary artery occlusion are likely to be missed during the early stages of myocardial infarction, resulting in delayed blood flow reconstruction, larger infarct size, and poorer prognosis [48]. In a case-control study, it was found that about half of the patients with NOAF had severe lesions of the RCA or LCX that were 25 times more severe than the lesions in those without AF [26]. Similarly, our study revealed that the lesions in the NOAF group were more concentrated in the RCA and LCX.

FIB is an acute-phase protein involved in systemic inflammation and plays a role in the normal physiological process of blood coagulation. The underlying pathophysiological mechanisms of cardiovascular risk mediated by FIB are multifaceted: [1] the formation of the thrombin substrate that is involved in the last step of the coagulation cascade; [2] improved speed and reactivity of platelet aggregation, maintaining the blood hypercoagulable state, and promoting the formation of atherosclerotic plaques and thrombi [49]; [3] regulation of endothelial function; [4] promoting the proliferation and migration of smooth muscle cells [50]. FIB and its degradation products participate in the inflammatory response of atherosclerosis by binding to the action sites of lymphocytes and endothelial cells [51]. In advanced atherosclerotic plaques, excessive accumulation of extracellular matrix proteins, such as FIB, promotes the formation of a fibrous cap, leading to a localized swelling of the vascular wall and further limiting the internal diameter of blood vessels that represents the core process of atherosclerotic lesions [52]. FIB is involved in the entire process of atherosclerosis. The serum FIB level is an independent predictor of death or nonfatal reinfarction in patients with NSTEMI treated with PCI, and its accuracy is similar to that of the GRACE scoring system [53]. FIB was also independently associated with 2-year all-cause and cardiac mortality in patients with CAD treated with PCI [11]. In a meta-analysis of coagulation and AF, there was a significant association between FIB levels and the incidence of AF; however, other cardiovascular risk factors, such as BMI or complications, weakened this association [54]. The association between FIB levels and NOAF incidence after PCI has not been studied.

ALB has multiple physiological properties such as antiinflammation, antioxidation, anticoagulation, antiplatelet aggregation, and maintenance of capillary membrane stability [55]. Decreased serum ALB levels increase the activity of vascular cell adhesion molecule-1 in endothelial cells and reduce inflammation, resulting in vascular endothelial injury. In addition, a decrease in serum ALB levels can increase the concentration of free lysophosphatidylcholine, stimulate the synthesis of lipids and coagulation factors, and increase blood viscosity, resulting in hyperlipidemia and a hypercoagulable state [56]. Endothelial injury and a hypercoagulable state further promote the formation of atherosclerotic plaques and thrombosis. Lower serum ALB concentrations have been associated with an increased risk of cardiovascular death [57]. Low serum ALB levels are associated with adverse cardiac events in patients with CAD after PCI [14]. Another study found that ALB levels are independently associated with a long-term risk of AF in octogenarians after the implantation of dual chamber permanent pacemakers [58].A community atherosclerosis risk study reported an independent, inverse, and linear correlation between serum ALB levels and the occurrence of AF events, although the causal effect was unclear [59]. As such, ALB may serve as an excellent prognostic factor, but therapeutics targeted at increasing ALB are unlikely to prevent AF effectively. Further research is needed to understand the relationship between ALB and AF or the role of additional factors that amplify the underlying causal relationship between ALB and AF.

Coagulation and inflammation are the chronic bases of atherosclerosis and can be activated by the same type of stimulus, including acute stress, surgery, and cross-linking in time and space in the same tissue [7]. The synergistic effect between inflammation and coagulation places the body in a state of high coagulation and low fibrinolysis, and this may lead to systemic and local biochemical changes and remodeling of the atrial structure, resulting in AF. Inflammation and immune thrombosis may also be associated with AF through other cardiovascular risk or susceptibility factors, such as coronary heart disease or heart failure [60].

The FAR, a new marker of inflammation and the pre-embolization state, can predict not only the prognosis of many tumors, such as ovarian cancer [61], but also the occurrence of thromboembolism-related complications [62]. So far, a large number of studies have been conducted to explore the relationship between FAR and CAD. Regardless of the type of AMI, FAR can not only assess the severity of the lesion but also evaluate the prognosis. It is worth emphasizing that in assessing the severity of CAD, FAR is significantly correlated with the SYNTAX score. In predicting prognosis and risk stratification, FAR can improve deficiencies in GRACE scores, making predictions more sensitive [16, 63, 64]. These studies also emphasize that FAR performs better than FIB or ALB alone. Our study also demonstrated that FAR was more effective than FIB, ALB, and other indicators in predicting the occurrence of hospital-acquired NOAF after PCI. FAR is a simple and easy-to-assess serum biological indicator. The findings of our study will help stratify the risk of AMI in patients receiving PCI and identify the risk of AF in patients as early as possible.

In patients with AMI, dual antiplatelet therapy (DAPT) using aspirin and P2Y12 inhibitors can significantly reduce the occurrence of ischemic events [65]. In patients with AF, oral anticoagulant (OAC) treatment is effective in preventing stroke and other thromboembolic events [66]. However, triple therapy ( TAT) combined with DAPT and OAC usually increases the risk of bleeding [67], especially when this regimen is used for a long period [68]. However, in which TAT was discontinued in all patients at 6-months, found no bleeding excess. Therefore, whether the excessive bleeding complications observed in a few studies should not be attributed to TAT itself, the duration and or composition of TAT should be further studied. This increase in bleeding risk is considered positively correlated with mortality [69, 70], making the clinical treatment of patients with AMI-NOAF more challenging. Moreover, in our study, most patients with NOAF were transient and recoverable, whereas some patients developed persistent AF and required drugs to control the ventricular rate and restore sinus rhythm. Related studies have also found that transient NOAF is associated with poor clinical outcomes and is an independent predictor of adverse cardio-cerebrovascular events and death in patients with AMI [71]. However, there is no consensus on whether these patients should be treated with anticoagulants.

Currently, artificial intelligence (AI) and machine learning (ML) have shown their effectiveness in CAD and AF management, including risk assessment, diagnosis, choice of therapy, remote monitoring, etc., for patients [72]. AI algorithms are capable of examining vast volumes of patient data, including medical history, laboratory findings, imaging investigations, and genetic information. We found that NOAF after PCI was mostly transient and not easily detected viatelemetric ECG and 24-h Holter monitoring. Fortunately, an AI-enabled ECG algorithm can identify patients with AF during sinus rhythm [73]. We hope that AI can also be used to predict NOAF after PCI, enabling risk assessment of such patients before surgery or proposing more effective intervention methods for such patients.

At present, it is not clear whether NOAF is a causative factor or a concomitant manifestation of poor prognosis. Further research is required to clarify the relationship between NOAF and poor prognosis after PCI. However, the benefit of the early identification of the risk of NOAF in patients is clear. As an early availability biological indicator, FAR shows good predictive ability for NOAF risk.

This study has some limitations that should be considered when interpreting the results. First, this was a single-center, retrospective, observational study with a small sample size. Identified and undetermined confounding factors may have affected the results. Therefore, a multicenter study with a larger sample size is needed to validate our findings. Second, the small number of newly diagnosed patients with AF studied by us may have limited the number of independent predictive factors identified and the consistency of the results. Third, some patients may have had unobserved or unrecorded paroxysmal AF. Finally, we focused only on the occurrence of NOAF during hospitalization and did not investigate the impact of improved FAR on future outcomes.

## Conclusions

Preoperative FAR proved to be an independent predictor of NOAF during hospitalization in patients with AMI after PCI. Therefore, it may be useful for the stratification of early risk in patients with AMI after PCI.

### Electronic supplementary material

Below is the link to the electronic supplementary material.

Supplementary Material 1

## Data Availability

The datasets generated and/or analyzed during the current study are not publicly available due to the restrictions of human genetics data policy of the Human Ethics Committee of Zhejiang Provincial People’s Hospital, but are available from the corresponding author on reasonable request.

## Declarations

### Authors’ information

Jiaqi Bao: Master’s degree student at Zhejiang Chinese Medical University.

Zhicheng Gao: Master’s degree student at Zhejiang Chinese Medical University.

Yilan Hu: Master’s degree student at Zhejiang Chinese Medical University.

Wenquan Liu: Master’s degree student at Jinzhou Medical University.

Lifang Ye: Master of Medicine, attending physician, Department of Cardiology, Zhejiang People’s Hospital.

Lihong Wang: Doctor of Medicine, Professor, Chief Physician, Doctoral Supervisor, Director of Cardiovascular Medicine Department of Zhejiang Provincial People’s Hospital.

## Abbreviations

NOAF: New-onset atrial fibrillation
PCI: Percutaneous coronary intervention
AMI: Acute myocardial infarction
FAR: Fibrinogen-to-albumin ratio
CAR: C-reactive protein-to-albumin ratio
FIB: Fibrinogen
CAD: Coronary artery disease
ALB: Albumin
AF: Atrial fibrillation
STEMI: ST-segment elevation myocardial infarction
ECG: Electrocardiogram
CABG: Coronary artery bypass grafting
BMI: Body mass index
eGFR: Estimated glomerular filtration rate
BNP: B-type natriuretic peptide
LA: Left atrial diameter
LVEF: Left ventricular ejection fraction
LM: Left main artery
LAD: Left anterior descending artery
LCX: Left circumflex artery
RCA: Right coronary artery
VIF: Variance expansion factor
ROC: Receiver operating characteristic
WBC: White blood cells
Scr: Serum creatinine
OR: Odds ratio
CI: Confidence interval
AUC: Area under the curve

## References

[1] Schmitt J, Duray G, Gersh BJ, Hohnloser SH (2009). Atrial fibrillation in acute myocardial infarction: a systematic review of the incidence, clinical features and prognostic implications. Eur Heart J.

[2] Luo J, Li H, Qin X, Liu B, Zhao J, Maihe G (2018). Increased risk of ischemic stroke associated with new-onset atrial fibrillation complicating acute coronary syndrome: a systematic review and meta-analysis. Int J Cardiol.

[3] Jabre P, Roger VL, Murad MH, Chamberlain AM, Prokop L, Adnet F (2011). Mortality associated with atrial fibrillation in patients with myocardial infarction: a systematic review and meta-analysis. Circulation.

[4] El-Battrawy I, Borggrefe M, Akin I (2017). Atrial fibrillation as a risk factor for worse outcome in acute coronary syndrome. Int J Cardiol.

[5] Grönefeld GC, Mauss O, Li YG, Klingenheben T, Hohnloser SH (2000). Association between atrial fibrillation and appropriate implantable cardioverter defibrillator therapy: results from a prospective study. J Cardiovasc Electrophysiol.

[6] Madsen JM, Jacobsen MR, Sabbah M, Topal DG, Jabbari R, Glinge C (2021). Long-term prognostic outcomes and implication of oral anticoagulants in patients with new-onset atrial fibrillation following st-segment elevation myocardial infarction. Am Heart J.

[7] Davalos D, Akassoglou K (2012). Fibrinogen as a key regulator of inflammation in disease. Semin Immunopathol.

[8] Kryczka KE, Kruk M, Demkow M, Lubiszewska B. Fibrinogen and a Triad of Thrombosis, Inflammation, and the Renin-Angiotensin System in Premature Coronary Artery Disease in Women: A New Insight into Sex-Related Differences in the Pathogenesis of the Disease. Biomolecules. 2021;11(7). 10.3390/biom11071036 PMC8301902 34356659

[9] Ang L, Behnamfar O, Palakodeti S, Lin F, Pourdjabbar A, Patel MP et al. Elevated baseline serum fibrinogen: Effect on 2-Year major adverse Cardiovascular events following percutaneous coronary intervention. J Am Heart Assoc. 2017;6(11). 10.1161/JAHA.117.006580 PMC5721757 29151032

[10] Lowe GD (1995). Fibrinogen and cardiovascular disease: historical introduction. Eur Heart J.

[11] Yuan D, Jiang P, Zhu P, Jia S, Zhang C, Liu Y (2021). Prognostic value of fibrinogen in patients with coronary artery disease and prediabetes or diabetes following percutaneous coronary intervention: 5-year findings from a large cohort study. Cardiovasc Diabetol.

[12] Jiang P, Gao Z, Zhao W, Song Y, Tang X-F, Xu J-J (2019). Relationship between fibrinogen levels and cardiovascular events in patients receiving percutaneous coronary intervention: a large single-center study. Chin Med J (Engl).

[13] Phillips A, Shaper AG, Whincup PH (1989). Association between serum albumin and mortality from cardiovascular disease, cancer, and other causes. Lancet.

[14] Wada H, Dohi T, Miyauchi K, Shitara J, Endo H, Doi S (2017). Impact of serum albumin levels on long-term outcomes in patients undergoing percutaneous coronary intervention. Heart Vessels.

[15] Barbano B, Gigante A, Amoroso A, Cianci R (2013). Thrombosis in nephrotic syndrome. Semin Thromb Hemost.

[16] Xiao L, Jia Y, Wang X, Huang H. The impact of preoperative fibrinogen-albumin ratio on mortality in patients with acute ST-segment elevation myocardial infarction undergoing primary percutaneous coronary intervention. Clin Chim Acta. 2019;493. 10.1016/j.cca.2019.02.018 30796900

[17] Güney B, Taştan Y, Doğantekin B, Serindağ Z, Yeniçeri M, Çiçek V (2021). Predictive value of CAR for In-Hospital mortality in patients with COVID-19 pneumonia: a retrospective cohort study. Arch Med Res.

[18] Çinier G, Hayıroğlu M, Kolak Z, Tezen O, Yumurtaş A, Pay L (2021). The value of C-reactive protein-to-albumin ratio in predicting long-term mortality among HFrEF patients with implantable cardiac defibrillators. Eur J Clin Invest.

[19] Myocardial infarction redefined, –, a consensus document of The Joint European Society (2000). Of Cardiology/American College of Cardiology Committee for the redefinition of myocardial infarction. Eur Heart J.

[20] Kirchhof P, Benussi S, Kotecha D, Ahlsson A, Atar D, Casadei B (2016). 2016 ESC Guidelines for the management of atrial fibrillation developed in collaboration with EACTS. Eur Heart J.

[21] Zhang E-Y, Cui L, Li Z-Y, Liu T, Li G-P (2015). High Killips Class as a predictor of new-onset Atrial Fibrillation following Acute myocardial infarction: systematic review and Meta-analysis. Chin Med J (Engl).

[22] Fauchier L, Bisson A, Bodin A, Herbert J, Angoulvant D, Danchin N (2021). Outcomes in patients with acute myocardial infarction and new atrial fibrillation: a nationwide analysis. Clin Res Cardiol.

[23] Asanin M, Perunicic J, Mrdovic I, Matic M, Vujisic-Tesic B, Arandjelovic A (2005). Prognostic significance of new atrial fibrillation and its relation to heart failure following acute myocardial infarction. Eur J Heart Fail.

[24] Clark DM, Plumb VJ, Epstein AE, Kay GN (1997). Hemodynamic effects of an irregular sequence of ventricular cycle lengths during atrial fibrillation. J Am Coll Cardiol.

[25] Ulus T, Isgandarov K, Yilmaz AS, Vasi I, Moghanchızadeh SH, Mutlu F (2018). Predictors of new-onset atrial fibrillation in elderly patients with acute coronary syndrome undergoing percutaneous coronary intervention. Aging Clin Exp Res.

[26] Alasady M, Abhayaratna WP, Leong DP, Lim HS, Abed HS, Brooks AG (2011). Coronary artery disease affecting the atrial branches is an independent determinant of atrial fibrillation after myocardial infarction. Heart Rhythm.

[27] Hu Y-F, Chen Y-J, Lin Y-J, Chen S-A (2015). Inflammation and the pathogenesis of atrial fibrillation. Nat Rev Cardiol.

[28] Celik S, Erdöl C, Baykan M, Kaplan S, Kasap H. Relation between paroxysmal atrial fibrillation and left ventricular diastolic function in patients with acute myocardial infarction. Am J Cardiol. 2001;88(2). 10.1016/s0002-9149(01)01611-3 11448413

[29] Nagahama Y, Sugiura T, Takehana K, Hatada K, Inada M, Iwasaka T (1998). The role of infarction-associated pericarditis on the occurrence of atrial fibrillation. Eur Heart J.

[30] He J, Yang Y, Zhang G, Lu X-H (2019). Clinical risk factors for new-onset atrial fibrillation in acute myocardial infarction: a systematic review and meta-analysis. Med (Baltim).

[31] Shiyovich A, Axelrod M, Gilutz H, Plakht Y (2019). Early Versus Late New-Onset Atrial Fibrillation in Acute myocardial infarction: differences in clinical characteristics and predictors. Angiology.

[32] Wu N, Li J, Xu X, Yuan Z, Yang L, Chen Y (2023). Prediction model of New Onset Atrial Fibrillation in patients with Acute Coronary Syndrome. Int J Clin Pract.

[33] Wanahita N, Messerli FH, Bangalore S, Gami AS, Somers VK, Steinberg JS (2008). Atrial fibrillation and obesity–results of a meta-analysis. Am Heart J.

[34] Gruberg L, Weissman NJ, Waksman R, Fuchs S, Deible R, Pinnow EE (2002). The impact of obesity on the short-term and long-term outcomes after percutaneous coronary intervention: the obesity paradox?. J Am Coll Cardiol.

[35] Park S-J, Ha KH, Kim DJ (2020). Body mass index and cardiovascular outcomes in patients with acute coronary syndrome by diabetes status: the obesity paradox in a korean national cohort study. Cardiovasc Diabetol.

[36] Lau DCW, Dhillon B, Yan H, Szmitko PE, Verma S (2005). Adipokines: molecular links between obesity and atheroslcerosis. Am J Physiol Heart Circ Physiol.

[37] Uretsky S, Messerli FH, Bangalore S, Champion A, Cooper-Dehoff RM, Zhou Q (2007). Obesity paradox in patients with hypertension and coronary artery disease. Am J Med.

[38] Miller M. Is hypertriglyceridaemia an independent risk factor for coronary heart disease? The epidemiological evidence. Eur Heart J. 1998;19 Suppl H:H18–H22. 9717060

[39] Kopin L, Lowenstein C, Dyslipidemia (2017). Ann Intern Med.

[40] Liu L, Liu X, Ding X, Chen H, Li W, Li H (2023). Lipid levels and New-Onset Atrial Fibrillation in patients with Acute myocardial infarction. J Atheroscler Thromb.

[41] Lopez FL, Agarwal SK, Maclehose RF, Soliman EZ, Sharrett AR, Huxley RR (2012). Blood lipid levels, lipid-lowering medications, and the incidence of atrial fibrillation: the atherosclerosis risk in communities study. Circ Arrhythm Electrophysiol.

[42] Hissa B, Oakes PW, Pontes B, Ramírez-San Juan G, Gardel ML (2017). Cholesterol depletion impairs contractile machinery in neonatal rat cardiomyocytes. Sci Rep.

[43] Güzel T, Aktan A, Demir M, Özbek M, Aslan B. Relationship between contrast-induced nephropathy and long-term mortality after percutaneous coronary intervention in patients with chronic coronary total occlusion. Rev Assoc Med Bras (1992). 2022;68(8):1078-83. 10.1590/1806-9282.20220283 PMC9574976 36000604

[44] Galvão Braga C, Ramos V, Vieira C, Martins J, Ribeiro S, Gaspar A (2014). New-onset atrial fibrillation during acute coronary syndromes: predictors and prognosis. Rev Port Cardiol.

[45] Aronson D, Mutlak D, Bahouth F, Bishara R, Hammerman H, Lessick J (2011). Restrictive left ventricular filling pattern and risk of new-onset atrial fibrillation after acute myocardial infarction. Am J Cardiol.

[46] Ravelli F, Allessie M (1997). Effects of atrial dilatation on refractory period and vulnerability to atrial fibrillation in the isolated Langendorff-perfused rabbit heart. Circulation.

[47] Halim SA, Clare RM, Newby LK, Lokhnygina Y, Schweiger MJ, Hof AW (2016). Frequency, clinical and angiographic characteristics, and outcomes of high-risk non-ST-segment elevation acute coronary syndromes patients with left circumflex culprit lesions. Int J Cardiol.

[48] Badings EA, Hermanides RS, The SHK, Dambrink J-HE, Rasoul S, Van Wijngaarden J (2018). Comparison of outcomes and intervention among patients with Non-ST-Segment elevation Acute myocardial infarction of those with a left Circumflex Versus those with a Non-Left Circumflex-Related coronary artery (from the ELISA-3 trial). Am J Cardiol.

[49] Tousoulis D, Papageorgiou N, Androulakis E, Briasoulis A, Antoniades C, Stefanadis C (2011). Fibrinogen and cardiovascular disease: genetics and biomarkers. Blood Rev.

[50] Koenig W (2003). Fibrin(ogen) in cardiovascular disease: an update. Thromb Haemost.

[51] Guo Y-H, Hernandez I, Isermann B, Kang T-b, Medved L, Sood R (2009). Caveolin-1-dependent apoptosis induced by fibrin degradation products. Blood.

[52] Hansson GK, Hermansson A (2011). The immune system in atherosclerosis. Nat Immunol.

[53] Song J, Yu T, Sun Z, Li Z, He D, Sun Z (2020). Comparison of prognostic significance between serum fibrinogen and Global Registry of Acute coronary events score for prognosis of patients with non-ST-elevation acute coronary syndromes undergoing percutaneous coronary intervention. Coron Artery Dis.

[54] Tilly MJ, Geurts S, Pezzullo AM, Bramer WM, de Groot NMS, Kavousi M (2023). The association of coagulation and atrial fibrillation: a systematic review and meta-analysis. Europace.

[55] Arques S. Human serum albumin in cardiovascular diseases. Eur J Intern Med. 2018;52. 10.1016/j.ejim.2018.04.014 29680174

[56] Joles JA, Willekes-Koolschijn N, Koomans HA (1997). Hypoalbuminemia causes high blood viscosity by increasing red cell lysophosphatidylcholine. Kidney Int.

[57] Danesh J, Collins R, Appleby P, Peto R (1998). Association of fibrinogen, C-reactive protein, albumin, or leukocyte count with coronary heart disease: meta-analyses of prospective studies. JAMA.

[58] Hayıroğlu M, Çınar T, Çinier G, Yüksel G, Pay L, Keskin K (2022). Cardiac variables associated with atrial fibrillation occurrence and mortality in octogenarians implanted with dual chamber permanent pacemakers. Aging Clin Exp Res.

[59] Liao L-Z, Zhang S-Z, Li W-D, Liu Y, Li J-P, Zhuang X-D (2020). Serum albumin and atrial fibrillation: insights from epidemiological and mendelian randomization studies. Eur J Epidemiol.

[60] Tilly MJ, Geurts S, Donkel SJ, Ikram MA, de Groot NMS, de Maat MPM et al. Immunothrombosis and new-onset atrial fibrillation in the general population: the Rotterdam Study. Clin Res Cardiol. 2022;111(1). 10.1007/s00392-021-01938-4 PMC8766396 34559294

[61] Chen W, Shan B, Zhou S, Yang H, Ye S (2022). Fibrinogen/albumin ratio as a promising predictor of platinum response and survival in ovarian clear cell carcinoma. BMC Cancer.

[62] Roth S, Jansen C, M’Pembele R, Stroda A, Boeken U, Akhyari P (2021). Fibrinogen-albumin-ratio is an independent predictor of thromboembolic complications in patients undergoing VA-ECMO. Sci Rep.

[63] Erdoğan G, Arslan U, Yenercağ M, Durmuş G, Tuğrul S, Şahin İ. Relationship between the fibrinogen-to-albumin ratio and SYNTAX score in patients with non-st-elevation myocardial infarction. Rev Invest Clin. 2021. 10.24875/RIC.20000534 33535227

[64] Çetin M, Erdoğan T, Kırış T, Özer S, Yılmaz AS, Durak H (2020). Predictive value of fibrinogen-to-albumin ratio in acute coronary syndrome. Herz.

[65] Levine GN, Bates ER, Bittl JA, Brindis RG, Fihn SD, Fleisher LA (2016). ACC/AHA Guideline focused update on duration of dual antiplatelet therapy in patients with coronary artery disease: a report of the American College of Cardiology/American Heart Association Task Force on Clinical Practice Guidelines: an update of the 2011 ACCF/AHA/SCAI Guideline for Percutaneous Coronary intervention, 2011 ACCF/AHA Guideline for coronary artery bypass graft surgery, 2012 ACC/AHA/ACP/AATS/PCNA/SCAI/STS Guideline for the diagnosis and management of patients with stable ischemic heart Disease, 2013 ACCF/AHA Guideline for the management of ST-Elevation myocardial infarction, 2014 AHA/ACC Guideline for the management of patients with Non-ST-Elevation Acute Coronary Syndromes, and 2014 ACC/AHA Guideline on Perioperative Cardiovascular evaluation and management of patients undergoing noncardiac surgery. Circulation.

[66] January CT, Wann LS, Calkins H, Chen LY, Cigarroa JE, Cleveland JC (2019). 2019 AHA/ACC/HRS focused update of the 2014 AHA/ACC/HRS Guideline for the management of patients with Atrial Fibrillation: a report of the American College of Cardiology/American Heart Association Task Force on Clinical Practice Guidelines and the heart rhythm society in collaboration with the Society of thoracic surgeons. Circulation.

[67] Sørensen R, Hansen ML, Abildstrom SZ, Hvelplund A, Andersson C, Jørgensen C (2009). Risk of bleeding in patients with acute myocardial infarction treated with different combinations of aspirin, clopidogrel, and vitamin K antagonists in Denmark: a retrospective analysis of nationwide registry data. Lancet.

[68] Gragnano F, Calabrò P, Valgimigli M (2019). Is triple antithrombotic therapy, or rather its duration and composition, the true culprit for the excess of bleeding events observed in patients with atrial fibrillation undergoing coronary intervention?. Eur Heart J.

[69] Valgimigli M, Costa F, Lokhnygina Y, Clare RM, Wallentin L, Moliterno DJ (2017). Trade-off of myocardial infarction vs. bleeding types on mortality after acute coronary syndrome: lessons from the thrombin receptor antagonist for clinical event reduction in Acute Coronary Syndrome (TRACER) randomized trial. Eur Heart J.

[70] Vaduganathan M, Harrington RA, Stone GW, Steg G, Gibson CM, Hamm CW (2018). Short- and long-term mortality following bleeding events in patients undergoing percutaneous coronary intervention: insights from four validated bleeding scales in the CHAMPION trials. EuroIntervention.

[71] Wi J, Shin D-H, Kim J-S, Kim B-K, Ko Y-G, Choi D (2016). Transient New-Onset Atrial Fibrillation is Associated with Poor Clinical Outcomes in patients with Acute myocardial infarction. Circ J.

[72] Hayıroğlu M, Altay S (2023). The role of Artificial Intelligence in Coronary Artery Disease and Atrial Fibrillation. Balkan Med J.

[73] Attia ZI, Noseworthy PA, Lopez-Jimenez F, Asirvatham SJ, Deshmukh AJ, Gersh BJ (2019). An artificial intelligence-enabled ECG algorithm for the identification of patients with atrial fibrillation during sinus rhythm: a retrospective analysis of outcome prediction. Lancet.

