# Supplementary material for: Serum fibrinogen-to-albumin ratio predicts new-onset atrial fibrillation risk during hospitalization in patients with acute myocardial infarction after percutaneous coronary intervention: a retrospective study
Source: BMC Cardiovasc Disord. 2023 Sep 1;23:432. doi: 10.1186/s12872-023-03480-9 (PMC10474692; doi:10.1186/s12872-023-03480-9)
Supplement: Supplementary file 1 — Supplementary Material 1 [file 12872_2023_3480_MOESM1_ESM.docx]

**Table** Linear relationship between FAR and NOAF

|  | **NOAF after PCI** | | | |
| --- | --- | --- | --- | --- |
|  | **Cases/Overall (％)** | **OR** | **95% CI** | **P-value** |
| **FAR median (range)** |  |  |  |  |
| 0.585 (＜0.67) | 4/167 (2.40％) | Reference | | |
| 0.737 (0.67-0.82) | 6/168 (3.57％) | 1.509 | 0.418-5.448 | **0.530** |
| 0.930 (0.82-1.07) | 12/168 (7.14％) | 3.135 | 0.990-9.926 | **0.052** |
| 1.371 (≥1.07) | 31/167 (18.56％) | 9.289 | 3.199-21.968 | **< 0.001** |
| **P for trend** | **< 0.001** | | | |

**Abbreviations:** AUC, area under the curve; CI, confidence interval; FAR, Fibrin/Albumin ratio; AMI, Acute myocardial infarction; STEMI, ST segment elevation myocardial infarction; NSTEMI, Non-ST segment elevation myocardial infarction; Fib, Serum fibrinogen; Alb, Serum albumin; BMI, Body mass index; Neu, Neutrophil count; Plt, platelet count; LA, Left atrium; LVEF, left ventricular ejection fraction
